# STIM1 Protein Activates Store-Operated Calcium Channels in Cellular Model of Huntington’s Disease

**Published:** 2014

**Authors:** V. A. Vigont, O. A. Zimina, L. N. Glushankova, J. A. Kolobkova, M. A. Ryazantseva, G. N. Mozhayeva, E. V. Kaznacheyeva

**Affiliations:** Institute of Cytology, Russian Academy of Sciences, Tikhoretsky pr., 4, St. Petersburg, 194064, Russia

**Keywords:** Huntington’s disease, calcium, neurodegeneration, SOC, STIM1

## Abstract

We have shown that the expression of full-length mutated huntingtin in human neuroblastoma cells (SK-N-SH) leads to an abnormal increase in calcium entry through store-operated channels. In this paper, the expression of the N-terminal fragment of mutated huntingtin (Htt138Q-1exon) is shown to be enough to provide an actual model for Huntington’s disease. We have shown that Htt138Q-1exon expression causes increased store-operated calcium entry, which is mediated by at least two types of channels in SK-N-SH cells with different reversal potentials. Calcium sensor, STIM1, is required for activation of store-operated calcium entry in these cells. The results provide grounds for considering the proteins responsible for the activation and maintenance of the store-operated calcium entry as promising targets for developing novel therapeutics for neurodegenerative diseases.

## INTRODUCTION

Abnormal calcium signaling has been detected in many diseases; in particular, destabilization of calcium ion channels of different types is associated with pathologies such as diabetes mellitus [1] or amyotrophic lateral sclerosis [2]. Many studies have demonstrated the involvement of impaired calcium signaling processes in neurodegeneration [3, 4].

Huntington’s disease (HD) is an autosomal dominant neurodegenerative disease caused by an increased number of repeats encoding glutamine in the first exon of protein-coding in the huntingtin gene. The length of the polyglutamine repeat normally does not exceed 35 residues; in the case of the disease, the length of repeats can reach up to 90 or more glutamine residues. [5] HD affects striatal neurons first.

In cells, huntingtin acts as an adapter protein that provides co-localization of the proteins interacting with it and helps these proteins to perform their functions. Many proteins interact with huntingtin, their function varying from vesicular transport and endocytosis to the regulation of transcription and apoptosis [6].

One of the toxic functions of mutant huntingtin is destabilization of calcium signaling. It was previously shown that mutant huntingtin is capable of binding directly to the C-terminus of the inositol 1,4,5-trisphosphate 1 receptor (IP3R1). Such binding increases IP3R1 sensitivity to its ligand, which may activate the receptor and deplete the intracellular calcium stores in response to IP3 basal concentration in cytosol [7]. It was also shown that expression of mutant huntingtin increases the function of NR2B-containing NMDA receptors [8] and affects the voltage-gated calcium channels [9]. All the pathways mentioned above cause an increased concentration of calcium ions in the cytosol and, as a consequence, abnormal accumulation of calcium in mitochondria [10, 11], activation of calpains [12], pathological initiation of calcium-dependent signaling pathways, and apoptotic activity of neuronal degeneration.

Previously, we detected an abnormal activation of store-operated calcium channels in human SK-N-SH neuroblastoma cells, expressing the full-length mutant huntingtin protein for the modeling of HD [13]. In addition, we demonstrated that the store-operated calcium entry can be considered as a potential target for therapeutic intervention in the development of new approaches for treating HD. The hyperactivation of store-operated calcium entry in striatal neurons isolated from YAC128 mice, used as a model for HD, was demonstrated using the fluorescent method [14].

It is believed that HD is associated with cleavage of the N-terminal fragment of mutant huntingtin that contains the polyglutamine tract and is encoded by the first exon of the huntingtin gene. This process is accompanied by the accumulation of the cleaved fragments in the nucleus, whereas the wild-type huntingtin is mainly localized in cytosol [15, 16]. It was also shown that expression of only the N-terminal fragment of pathogenic huntingtin is sufficient for an increased sensitivity of IP_3_R1 to IP_3_ [7].

Therefore, the goal of our study was to investigate the changes in the operation of store-operated calcium channels in SK-N-SH cells expressing the first exon of the pathological huntingtin gene with 138 glutamine residues in the tract (Htt138Q-1exon) and to identify the role of the STIM1 protein in the activation of these channels.

## MATERIALS AND METHODS

**Cells**

Human neuroblastoma SK-N-SH cells from the cell culture collection of the Institute of Cytology, RAS, were cultured on a DMEM medium supplemented with a 10% fetal calf serum and an antibiotic (80 mg/ml gentamicin). The cells were plated on 3 × 3 mm glass cover-slip fragments 2–3 days prior to the experiment. The cover glasses were coated with a 0.01% polylysine for better cell adhesion.

**Infection of cells, transfection, and RNA interference**

A shuttle vector encoding the N-terminal fragment of the Htt138Q-1exon protein (270 amino acids) conjugated with the HA-tag, HIV-1-8.9 (Δ8.9)packing vector and VSVG plasmids encoding the surface glycoproteins of the viral particle were kindly provided by Prof. I. B. Besprozvanny (UT Southwestern Medical Center, USA). Virus Lenti-Htt138Q-1exon was obtained by co-transfection of the shuttle vector with the HΔ8.9-packing vector and VSVG plasmids encoding the surface glycoproteins in the packing HEK293T cell line. Petri dishes with the cells were incubated for 24 h at 37 °C and then for 72 h at 32 °C after the addition of the transfection solution to the medium. During this time, the packed viruses were excreted by the cells in the medium. The medium with viruses was filtered (O 0.45 μm) after incubation, immediately frozen in liquid nitrogen, and stored at –80 °C.

Immunostaining with anti-HA-tag antibodies was used to determine the virus titer. The proportion of infected cells out of the total number of cells on the glass was determined visually using a Pascal microscope. Having estimated the efficiency of the infection based on the proportion of luminous cells, we chose the ratio between the virus-containing medium and the culture medium with a minimal efficiency of 90%.

The cells were infected the following day after plating. The culture medium with the amount of lentivirus providing the minimal transfection efficiency of 90% was added to the cells.

In the control experiments, the cells were infected with an empty expression vector (control vector) (SIGMA, USA).

In experiments with the suppression of STIM1 expression in addition to infection of Lenti-Htt138Q-1exon cells, we used co-transfection with plasmid encoding the siRNA against STIM1 (SIGMA, USA) and plasmid encoding a green fluorescent protein (GFP), with a 3:1 ratio.

In the control experiments, we used a co-transfection of plasmid with siRNA without a specific target (control siRNA) (SIGMA, USA) and plasmids encoding GFP with a 3:1 ratio.

**Electrophoresis and immunoblotting**

The cells were grown in 50-mm Petri dishes. After transfection, the cells were lysed in a buffer solution containing 10 mM Tris-HCl pH 7.5, 150 mM NaCl, 1% Triton X-100, 1% NP40 (Nonidet P40, non-ionic detergent nonylphenyl*-*polyethylene glycol), 2 mM EDTA, 0.2 mM PMSF (phenylmethanesulfonyl fluoride, a serine protease inhibitor ) supplemented with protease inhibitors (PIC, Hoffmann-La Roche AG, Germany). Lysate proteins were separated by electrophoresis in a 8% polyacrylamide gel in a vertical chamber and transferred to a nitrocellulose membrane. Proteins were detected by immunoblotting using monoclonal anti-STIM1 antibodies (BD Bioscience, USA) diluted 1:250. The secondary antibodies were anti-mouse IgG produced in goat (1:30000). Proteins on immunoblots were detected using the Super Signal Chemiluminescent Substrate (PIERCE, USA). The experiments were repeated at least three times using different cell lysates. Specific monoclonal anti-α-tubulin antibodies (1:1000) (SIGMA, USA) were used to control equal loading. The percentage of protein content was compared using the standard program for comparing the color intensities of the scanned immunoblots.

**Electrophysiological measurements**

A patch clamp was used to detect ion currents for whole cell recordering [17]. All measurements were performed using an Axopatch 200B amplifier (Axon Instruments, USA). Resistance of the microelectrodes was 5–15 MΩ. Series resistance was not compensated. The signal was amplified and pre-filtered using a twopole Bessel filter built-in amplifier (cut off frequency 500 Hz). The signal was digitized at a frequency of 5000 Hz using an ADC board L305 (L-Card, Russia). The membrane potential was kept at –40 mV during the recording of integral currents within the cell. The membrane potential was changed to –100 mV (per 30 ms) periodically (every 5 s), and then the membrane potential was gradually changed to +100 mV at a constant rate of 1 mV/ms. The measurement interval was 0.5 mV. The recorded currents were normalized for cell capacity (10–30 pF). The records obtained prior to the activation of the investigated currents were used to subtract the leak current and currents via other channels.

**Solutions**

In the measurements made in the whole-cell configuration, the recording pipette solution contained (mM): 135 CsCl, 10 EGTA-Cs, 30 Hepes-Cs, 4.5 CaCl_2_, 1.5 MgCl_2_, 4 Na-ATP, 0.4 Na_2_-GTP (pCa7), pH 7.3. The extracellular solution contained (mM): 130 NMDG-Asp, 10 BaCl_2_, 20 Hepes-Cs, 0.01 nifedipine, pH 7.3.

Barium ions were selected as a current carrier to prevent calcium-dependent inactivation. Nifedipine was added to the solution of the experimental chamber in order to eliminate the possible contribution of integral L-type voltage-gated calcium channels to the inward current.

Thapsigargin (1 μM) was added to the extracellular solution to activate store-operated currents; the solution was supplied to the object by perfusion of the experimental chamber. The solution replacement time in the chamber was less than 1 s.

**Calculation**

Calculations of electrophysiological data and linearization of the current-voltage characteristics were performed using the OriginPro 8.0 software package.

## RESULTS AND DISCUSSION

To simulate HD, SK-N-SH human neuroblastoma cells were infected with a lentivirus containing the construct encoding the product of the first exon of the huntingtin gene with a polyglutamine tract consisting of 138 glutamine residues (Htt138Q-1exon).

Thapsigargin is an irreversible blocker of all SERCA (sarco/endoplasmic reticulum Ca_2_^+^-ATPase) isoforms that operate in the membranes of the endoplasmic reticulum (ER) as calcium pumps and control the pumping of calcium ions from the cytosol into the ER lumen. Thapsigargin (1 mM) was added to the solution to activate store-operated calcium channels. The recorded current can only be attributed to the operation of store-operated channels, since application of Thapsigargin leads to passive depletion of the stores and does not affect other cellular signaling pathways.

**Fig. 1:**
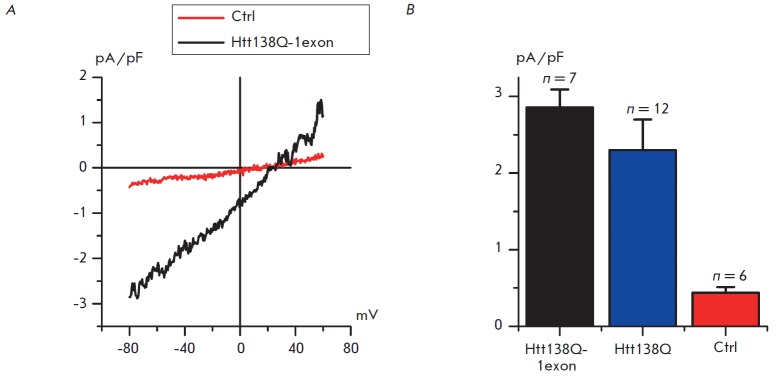
Effect of the lentiviral expression of Htt138Q-1exon on the level of store-operated calcium currents in SK-N-SH cells. A – the average I-V curves of currents evoked by passive depletion of calcium stores by 1 mM Thapsigargin in SKN- SH cells expressing the Htt138Q-1exon (*black line*), control SK-N-SH cells expressing the control empty vector (*red line*). I-V curves were recorded after full development of the store-operated currents. Each trace is an average based on the number of experiments as indicated in (*B*). B *– *The average amplitude of store-operated currents in control SKN- SH cells (*red filling*) and in SK-N-SH transfected with full-length Htt138Q (*blue filling*) or infected with Htt138Q-1exon (*black filling*). For all groups of cells, the amplitude was determined at a potential of –80 mV and plotted as a mean ± SE (n = number of experiments). *p * < 0.05

The analysis of electrophysiological experiments with the patch clamp technique in the whole-cell configuration, in response to the application of Thapsigargin (1 mM), demonstrated that store-operated entry of calcium was significantly higher in cells expressing the first exon of mutant huntingtin than in the control cells (ctrl) expressing the empty control vector (*Fig. 1A*). The amplitude of thapsigargin-induced currents in Htt138Q-1exon cells was 2.86 ± 0.24 pA/pF, whereas the amplitude of the same currents in the control cells was only 0.44 ± 0.07 pA/pF.

Based on a comparison with the data obtained earlier for SK-N-SH cells expressing full-length mutant huntingtin [13, 14], we could conclude that the expression of the full-length Htt138Q protein and the product of the first Htt138Q-1exon exon have almost the same effect on the level of store-operated calcium entry in SK-N-SH cells (*Fig. 1B*). The amplitude of store-operated calcium entry was 2.86 ± 0.24 pA/pF in SK-N-SH cells expressing the N-terminal fragment of pathogenic huntingtin; the amplitude for the expression of fulllength pathogenic huntingtin was 2.30 ± 0.40 pA/pF (*Fig. 1B*). The difference in the amplitudes of store-operated calcium entry for various HD models on SK-NSH cells was statistically insignificant (*p * < 0.05).

Thus, we have shown that the expression of the N-terminal fragment of mutant huntingtin in SK-NSH cells is an adequate model for investigating the impairment of store-operated calcium channels in HD.

Another objective of this study was to investigate the role of the STIM1 protein in the activation of store-operated calcium channels in the lentiviral model of HD.

STIM1 is an integral membrane protein of ER and the plasma membrane (PM) with a single transmembrane domain. It is believed that STIM1 is mainly localized in the ER membranes, and only about 15–25% of STIM1 is localized on the PM of the cells [18].

In the cells, STIM1 acts as a calcium sensor in the lumenal space of the ER and an activator of the store-operated channels of PM [18]. Normally, when the intracellular calcium stores are filled, the STIM1 protein is localized in the ER membrane in a non-oligomerized state. Calcium store depletion causes a number of conformational changes resulting in the clustering of STIM1 and its transport to the puncta region, adjacent to the PM [18]. The presence of the proline-rich domain in the C-terminal region of the STIM1 protein suggests the possibility of protein–protein interactions between individual STIM1 molecules, as well as interaction with other proteins. Moreover, the localization of STIM1 in ER membranes located in close proximity to the PM enables direct interaction between STIM1 in the ER membrane proteins with the proteins of PM.

Various channel-forming proteins and the plasmatic pools of STIM1 proteins are among the proteins that interact with the endoplasmic STIM1. It was shown that STIM1 interacts with the proteins responsible for store-operated calcium entry into different types of cells: TRPC proteins [19] and the Orai1 protein [20].

**Fig. 2:**
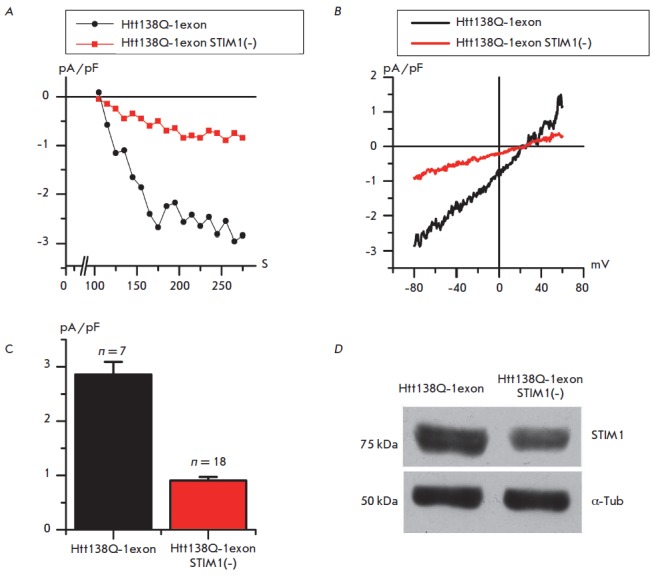
Effect of STIM1 suppression on the store-operated calcium currents in SK-N-SH Htt138Q-1exon cells. A – the amplitude of store-operated currents recorded in whole-cell experiments is shown as a function of time after the application of 1 mM thapsigargin to Htt138Q-1exon cells transfected with the control GFP protein (Htt138Q-1exon) (*black circles*) or transfected with GFP and siRNA against STIM1 (Htt138Q-1exon STIM1(–)) (*red squares*). The amplitude of the currents for all groups of cells was measured every 10 s at a potential of –80 mV. Data from representative experiments are shown. B – the average I-V curves of currents evoked by passive depletion of calcium stores with 1 mM thapsigargin in Htt138Q-1exon cells transfected with the control GFP protein (Htt138Q-1exon) (*black trace*) or transfected with GFP and siRNA against STIM1 (Htt138Q-1exon STIM1(–)) (*red trace*). I-V curves were recorded after full development of the store-operated currents. Each trace is an average based on a number of experiments as indicated in (*C*). C *– *the average amplitude of store-operated currents in Htt138Q-1exon cells transfected with the control GFP protein (Htt138Q- 1exon) (*black filling*) or transfected with GFP and siRNA against STIM1 (Htt138Q-1exon STIM1(–)) (*red filling*). For all groups of cells, the amplitude was determined at potential –80 mV and plotted as mean ± SE (n = number of experiments). p < 0.05. D **– **Western blot showing the levels of STIM1 expression in SK-N-SH Htt138Q-1exon cells transfected with the control GFP protein or transfected with GFP and siRNA against STIM1

Expression of STIM1 in Htt138Q-1exon cells was suppressed by small interfering RNA. The effectiveness of the suppression was confirmed by immunoblotting (*Fig. 2D*).

The results of electrophysiological experiments demonstrated that suppression of STIM1 leads to a marked decrease in the amplitude of thapsigargin- induced currents from 2.86 ± 0.24 pA/pF in Htt138Q-1exon cells to 0.91 ± 0.07 pA/pF in Htt138Q- 1exon STIM1(–) cells (*Fig. 2A, B, C*). Thus, we can conclude that the STIM1protein is a key element in the activation of the store-operated calcium response in Htt138Q-1exon cells.

**Fig. 3:**
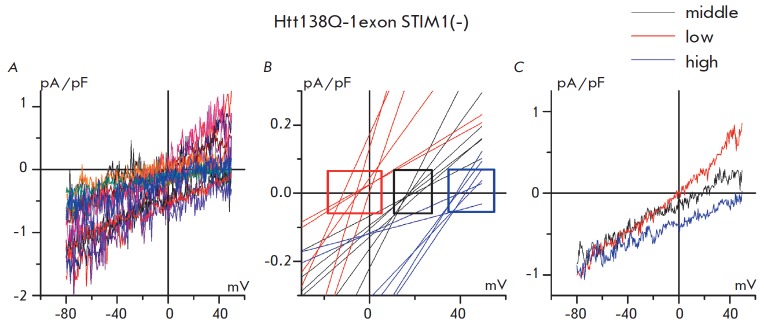
Store-operated calcium currents in SK-N-SH Htt138Q-1exon cells with suppression of the STIM1 protein. A **–** the non-averaged I-V curves of currents evoked by the passive depletion of calcium stores with 1 mM thapsigargin in Htt138Q-1exon cells transfected with the control GFP protein and siRNA against STIM1. I-V curves were recorded after full development of the store-operated currents. (*Each colored line represents an independent experiment*) B – the fragments of linear fit of I-V curves from the (*A*) panel with a low (*red lines*), medium (*black lines*), and high (*blue lines*) reversal potential. C **– **the average I-V curves of currents evoked by the passive depletion of calcium stores with 1 mM thapsigargin in Htt138Q-1exon STIM1(–) cells with a low (*red trace*), medium (*black trace*), and high (*blue trace*) reversal potential. I-V curves were recorded after full development of the store-operated currents

The reversal potential of the average current through the store-operated channels in Htt138Q-1exon STIM1(–) cells was not different from that in Htt138Q- 1exon cells (*Fig. 2B*). However, the plotting of the current-voltage characteristics (I-V curves) of separate experiments on the same graph demonstrated that store-operated currents in Htt138Q-1exon STIM1(–) cells have a wide range of different reversal potentials, indicating the different selectivity of the channels that mediate the store-operated current (*Fig. 3A*). A detailed analysis of the individual I-V curves of registered store-operated currents in Htt138Q-1exon STIM1(–) cells and their linearization showed that the reversal potentials of these currents can be divided into three different groups (*Fig. 3B, C*). Some I-V curves had low reversal potentials (less than 5 mV), while the second group had mid-level reversal potentials of about 20 mV, and finally, the third group had high reversal potentials (more than 35 mV). Thus, it becomes clear that more than one type of store-operated channels with different selectivities for Ca^2+^ are involved in the store-operated calcium entry in Htt138Q-1exon STIM1(–) cells. The amplitudes of the currents with high, medium, and low reversal potentials were similar to each other at a potential of –80 mV (*Fig. 3B*) and were 0.88 ± 0.20, 0.87 ± 0.17 and 1.00 ± 0.28 pA/pF, respectively.

**Fig. 4:**
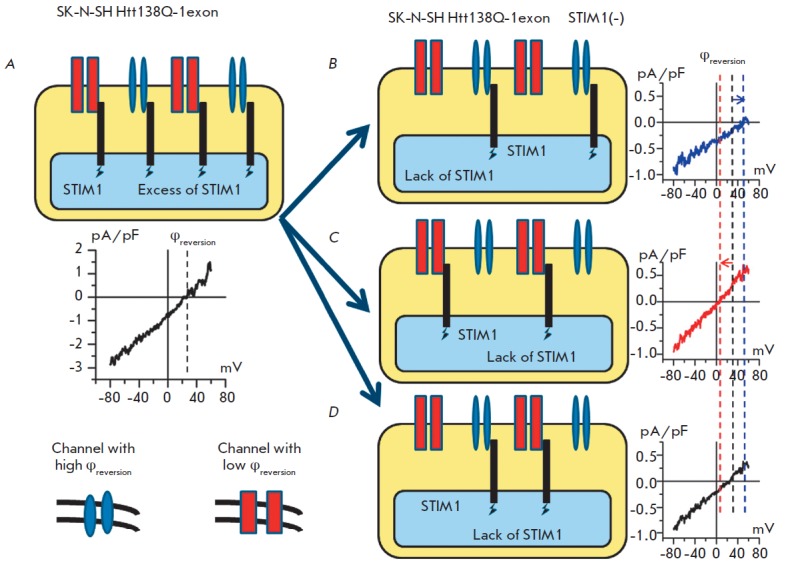
Possible pathway of activation of the store-operated calcium channels with different reversal potentials in SKN- SH Htt138Q-1exon STIM1 (-) cells. In this scheme, it is shown that activation of all types of store-operated channels could be observed under conditions of a large quantity of the STIM1 protein, resulting in average I-V curves of these currents with a medium reversal potential (*A*). Suppression of the STIM1 protein could lead to preferential activation of one type of store-operated channels with a high (*B*) or low (*C*) reversal potential. It also could lead to activation of an equal quantity of both channels with low and high reversal potentials (*D*). Channels with a high reversal potential are represented by blue ovals and a blue line on the plot. Channels with a low reversal potential are represented by red rectangles and a red line on the plot. Activation of both channel types is shown as black lines on the plots. Reversal potentials are shown by dotted lines of the corresponding colors

One hypothesis that explains these observations may be the assumption that there are two different types of channels in Htt138Q-1exon cells which are controlled by the store-operated mechanism and have similar amplitudes at a potential of –80 mV but different selectivities. In this case, when thapsigargin-induced currents are activated in Htt138Q-1exon cells, the I-V curves of integral currents are a superposition of two types of activated store-operated channels (*Fig. 2B*). As long as there is enough of the STIM1 protein responsible for the activation of store-operated entry in Htt138Q-1exon cells, the channels differing in their selectivity are activated to the same extent, producing averaged I-V curves with a reversal potential somewhere between the reversal potentials of each channel (*Fig. 2B, 4A*). When the STIM1 protein in Htt138Q-1exon STIM1(–) cells is suppressed, the equilibrium can be shifted toward the predominant activation of store-operated channels with a high (*Fig. 4B*) or low (*Fig. 4C*) reversal potential due to the lack of STIM1. Another possibility is the activation of an equal number of channels with different reversal potentials even when there is a lack of STIM1 (*Fig. 4D*), which can explain the experiments performed on Htt138Q- 1exon STIM1(–) cells, when average values of the reversal potential were observed.

This is only one possible explanation, and the actual chain of events could be much more complicated. For example, store-operated currents in Htt138Q-1exon cells may represent a superposition of not two, but three or more, channels. In particular, we demonstrated in previously published studies the existence of four types of channels with completely different biophysical properties, which can be activated by store-operated mechanisms in human embryonic kidney epithelium cells (HEK293 cell line) [21]. Similar results were obtained for A431 human epidermoid carcinoma cells [22-24].

## CONCLUSIONS

Thus, we have demonstrated that the expression of the N-terminal fragment of mutant huntingtin can effectively simulate the earlier described changes in store-operated calcium entry in human neuroblastoma cells. We have also found that the activation of store-operated calcium channels in SK-N-SH cells requires the presence of a calcium sensor; the STIM1 protein. Furthermore, we established that store-operated calcium entry in SK-N-SH cells simulating DH is controlled by at least two different types of channels.

## Abbreviations

HD: Huntington’s disease
I-V curves: current-voltage characteristic
PM: plasma membrane
ER: endoplasmic reticulum
Htt138Q-1exon: product of the 1st exon of the gene encoding mutated huntingtin or cells expressing this product
Htt138Q-1exon STIM1(-): Htt138Q-1exon cells with suppression of STIM1 protein
IP3: inositol 1,4,5-trisphosphate
IP3R1: receptor of inositol 1,4,5-trisphosphate 1
GFP: green fluorescent protein
SK-N-SH: human neuroblastoma cells
STIM1: stromal interaction molecule 1 (protein)
